# Evaluation of automatic tube current modulation of CT scanners using a dedicated and the CTDI dosimetry phantoms

**DOI:** 10.1002/acm2.13620

**Published:** 2022-06-09

**Authors:** Ioannis A. Tsalafoutas, Shady AlKhazzam, Huda AlNaemi, Mohammed Hassan Kharita

**Affiliations:** ^1^ Medical Physics Section OHS Department Hamad Medical Corporation Doha Qatar; ^2^ Weill Cornell Medicine‐Qatar Doha Qatar

**Keywords:** CT, CTDI phantom, image noise, quality control, tube current modulation

## Abstract

**Purpose:**

To investigate the operation principles of the automatic tube current modulation (ATCM) of a CT scanner, using a dedicated phantom and the CT dosimetry index (CTDI) phantom.

**Material and methods:**

The Mercury 4.0 phantom and three different configurations of the CTDI dosimetry phantom were employed. A frequently used clinical scanning protocol was employed as a basis for the acquisitions performed with all phantoms, using both scanning directions. Additional acquisitions with different pitch and examination protocols were performed with Mercury phantom, to further explore their effect on ATCM and the resulting image quality. Different software named DICOM Info Extractor, ImageJ, and imQuest, were used to derive CTDI_vol_ and table position, image noise, and water equivalent diameter (WED) of each phantom CT image, respectively. ImQuest was also used to derive the detectability index (d’) of five different materials (air, solid water, polystyrene, iodine, and bone) embedded in the Mercury phantom.

**Results:**

It was exhibited with all four phantoms that the scanning direction greatly affects the modulation curves. The fitting of the dose modulations curves suggested that for each table position what determines the CTDI_vol_ value is the WED values of the phantom structures laying ahead towards the scanning direction, for a length equal to the effective width of the X‐ray beam. Furthermore, it was also exhibited that ATCM does not fully compensate for larger thicknesses, since images of larger WED phantom sections present more noise (larger SD) in all four phantoms and in Mercury 4.0 phantom smaller detectability (d’).

**Conclusion:**

Mercury 4.0 is a dedicated phantom for a complete and in‐depth evaluation of the ATCM operation and the resulting image quality. However, in its absence, different CTDI configurations can be used as an alternative to investigate and comprehend some basic operation principles of the CT scanners’ ATCM systems.

## 1 INTRODUCTION

Modern CT scanners, usually referred to as multislice CT (MSCT) or multidetector CT (MDCT), are all equipped with automatic exposure control (AEC) systems. Traditionally, the primary goal of all AEC systems was to adapt the dose to obtain a uniform image quality irrespective of the scanned patient size.^1^, ^2^, ^3^ However, it could be probably more accurate to say that the primary goal of some AEC systems has been shifted to adapt the dose to the level required for a specific diagnostic task, considering the differences in size and density between different patients and between different anatomic regions within the same patient.^4^

In old CT scanners, a rough dose adaptation for different patient sizes was performed manually, by selecting appropriately the tube potential (kVp), tube current (mA), and exposure time per rotation (t), but none of these parameters could be varied during scanning. In modern MSCT scanners, a fixed value of rotation time and kVp is still used, though the kVp value can also be automatically selected prior to the CT scan when the Automatic Tube Voltage Selection (ATVS) feature is available. However, mA can be continuously adapted during scanning, allowing for the modulation of tube current, which effectively leads to the modulation of dose (i.e., the CTDI_vol_ values) for each patient, depending on the attenuation characteristics of each anatomical region scanned.^1^, ^2^, ^3^ For this reason, CT AEC systems are usually referred to as automatic tube current modulation (ATCM or TCM) systems. Though different CT manufacturers provide different methods and software to obtain the desired dose adaptation, a common characteristic is that the mapping of the attenuation characteristics of each patient or phantom is obtained using the scan projections radiographs (SPR) which precede the scans.^1^, ^2^, ^3^, ^4^

Several studies can be found in literature, in which various phantoms and methods have been used to evaluate the ATCM systems of various CT‐scanners.^1^, ^5^, ^6^, ^7^, ^8^, ^9^, ^10^ However, the importance of ATCM testing has been made more official with the publication of the AAPM TG233 report.^4^ The cited report analyzes in depth the specifics regarding ATCM systems, and reviews old and new image quality indices that can be used for image quality assessment in conjunction with the evaluation of ATCM systems. However, the AAPM TG233 report sets no performance guidelines nor pass‐fail criteria, but rather proposes assessment methods that pave the way for approaching the operational performance testing of new CT systems, not only for acceptance testing purposes but also for system commissioning and for determining how the system can be used most effectively in clinical practice. The assessment methods described in that report and the way that these can be utilized in the clinical context, should be still considered as work‐in‐progress. Nevertheless, these methods improve the understanding and utilization of the technologies available in each CT scanner and potentially will be used to compare CT scanners and examination protocol settings in terms of their performance in clinical conditions.^4^

**FIGURE 1:**
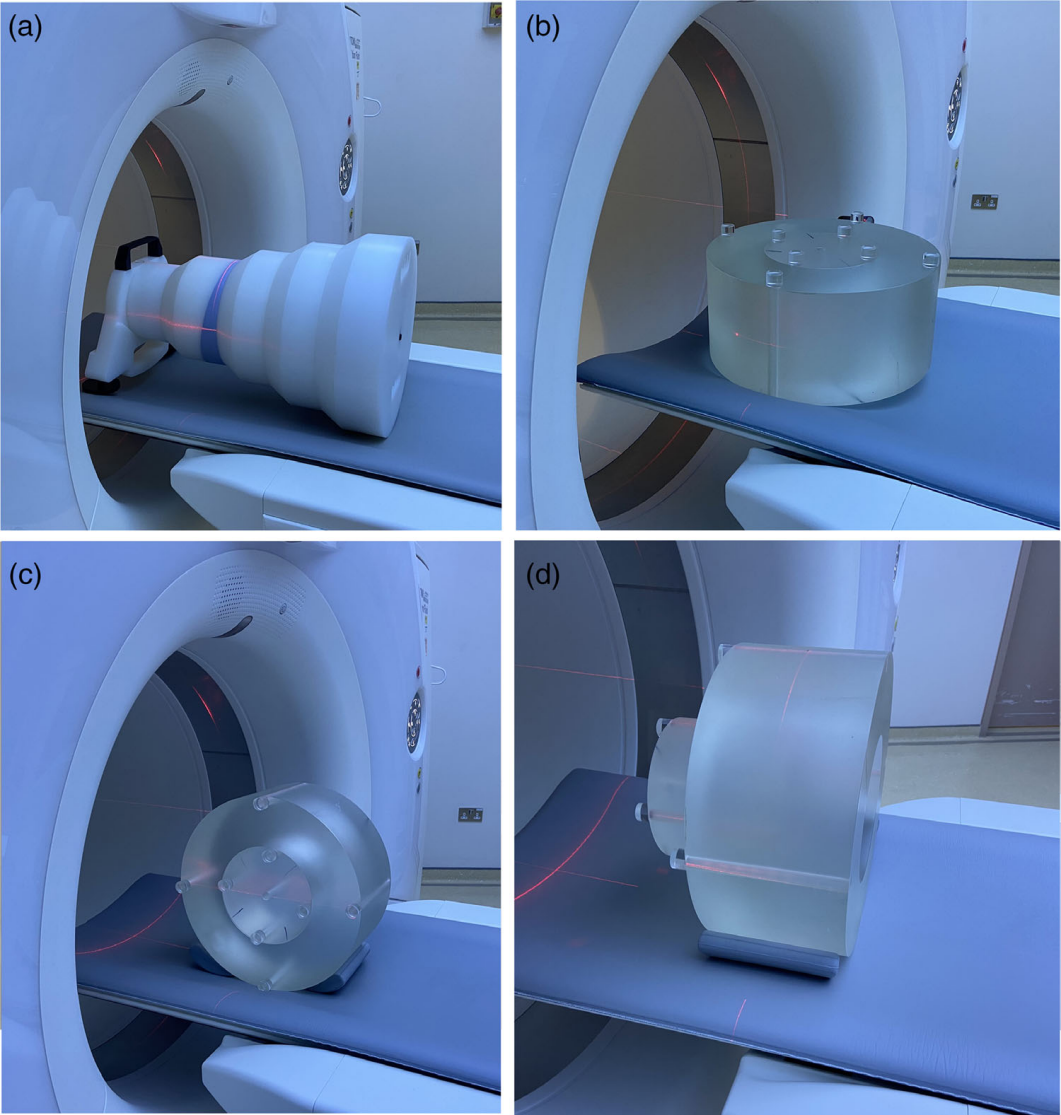
The phantoms used in the study and their set‐up on the CT table: (a) Mercury 4.0, (b) CTDI‐P1, (c) CTDI‐P2, and (d) CTDI‐T

The APPM TG233 report does not endorse any particular commercial phantom, but rather explores the utility of some commercially available phantoms, including the Gammex Mercury 4.0 phantom (shown in Figure 1a) and different configurations of the CTDI phantom.^4^ The first CTDI phantom configuration, is the body CTDI phantom with its facet planes positioned flat on the table (as shown in Figure 1b) and the second is the same phantom with its facet planes perpendicular to the CT table and parallel to the Z‐axis (as shown in Figure 1c). Both configurations have been used in a study recently published,^11^ soon after the publication of the AAPM TG233 report. The last configuration described in the AAPM TG233 report is a two‐section phantom created by the body and head CTDI phantoms, positioned one‐after the other, with their center on the same axis parallel to the Z‐axis.^4^ This configuration is a simpler version of the three‐section phantom, which can be created when the nested CTDI dosimetry phantom is available (comprised by a head phantom and a body ring) by offsetting the head part of the nested CTDI dosimetry phantom, halfway out of the body ring,^10^ as shown in Figure 1d. The resulting phantom has a head like section (PMMA cylinder of 16 cm in diameter), an abdomen like section (PMMA cylinder of 32 cm in diameter), and a lung like section (PMMA cylinder of 32 cm in diameter but with a 16 cm diameter cylindrical hole in its center), all having a 7.5 cm length.

In [blinded] 21 latest technology CT scanners from three different major manufacturers (Siemens, Philips and Canon) are currently installed, which employ different ATCM systems.^1^, ^2^, ^3^ Though in the context of the established quality assurance program, extensive QC tests are performed in all CT scanners based on the ACR recommendations,^12^ the operation of the ATCM systems has never been evaluated. To complement and evolve our QC procedures, the Gammex Mercury 4.0 phantom and the accompanying software package that allows for the automatic assessment of image quality (in line with the methodologies described in the AAPM TG233 report^4^) were recently procured. In the context of understanding the use of this phantom and the AAPM TG233 methodology, the ATCM performance of a single scanner was evaluated in terms of both dose and image quality adaptation.

Given the fact that the Mercury 4.0 is a relatively large, heavy, and expensive phantom that cannot be afforded by many medical physicists around the world, the proposed alternatives of CTDI dosimetry phantom were also utilized for ATCM performance evaluation of the same CT scanner in order to check whether, in absence of the Mercury phantom, the CTDI phantom can partly substitute it for the evaluation of ATCM systems.

## 2 MATERIALS AND METHODS

The Mercury 4.0 phantom and three different configurations of the CTDI dosimetry phantom were used to evaluate the ATCM system of a Siemens Somatom Definition Flash CT scanner. The main material of the Mercury 4.0 phantom is virgin ultra‐high molecular weight polyethylene (density = 0.93 g/cm^3^ and CT# = −90 HU) and is made up of five cylinders and four cone‐rings (tapered transitional sections between the cylinders) whose dimensions are described in Table 1. The five cylinders of the phantom contain five rods (targets) of the same diameter (2.5 cm) and length (3 cm), positioned at a distance 4.5 cm around the phantom's central axis. These are constructed by HE CT Solid Water® (CT# = 0 HU), bone (CT# = 910 HU), polystyrene (CT# = −40 HU), iodine (10 mg/ml) (CT# = 245 HU) and air (CT# = −985 HU). This phantom was positioned with its smallest diameter closest to the CT gantry, as shown in Figure 1a.

**TABLE 1:** Description of the Mercury 4.0 phantom. The water equivalent diameter was calculated by the software (imQuest v 7.1), which is used for the automatic evaluation of image quality

Phantom Regions	Shape and number	Diameter (cm)	Length^a^ (cm)	WED (cm) [Nominal]
Section 0	Handle	–	–	max 25.5
Section 1	Cylinder #1	16	7	16.5
Section 2	Cone ring #1	16–21	4	16.5–21
Section 3	Cylinder #2	21	9	21 (21.8^b^)
Section 4	Cone ring #2	21–26	4	21.5–25
Section 5	Cylinder #3	26	6	25.6
Section 6	Cone ring#3	26–31	4	25.4–29.8
Section 7	Cylinder #4	31	6	30.5
Section 8	Cone ring #4	31–36	4	30.5–35.1
Section 9^c^	Cylinder #5	36	7	35.2

^a^ 3 cm with five rods and the rest uniform.

^b^ Maximum WED observed at the region of the solid water ramp used for z‐axis resolution measurements.

^c^ A handgrip is engraved within the phantom.

The first CTDI phantom configuration used in this study (henceforth referred to as CTDI‐P1) is shown in Figure 1b and the second configuration (henceforth referred to as CTDI‐P2) is shown in Figure 1c. For different Z‐axis values, the CTDI‐P1 configuration exhibits varying thickness in the X‐axis and constant thickness in the Y‐axis (15 cm), while the CTDI‐P2 exhibits varying thickness in the Y‐axis and constant thickness in the X‐axis (15 cm). The third CTDI phantom configuration used in this study is the three‐section phantom (henceforth referred to as CTDI‐T), shown in Figure 1d.

All phantoms were initially scanned utilizing the most often used clinical protocol for helical scanning (Thorax CT‐Chest Navigation) and two different scanning directions, that is, from head‐to‐feet (HF) and from feet‐to‐head (FH). The standard collimation of 64 × 0.6 mm was used, with a selected pitch of 0.8 and a planned scan length that was extending beyond the phantom boundaries by about 4 cm (roughly equal to the total beam width). It must be noted that the SPRs on which the scans were designed, were extending at least 10 cm beyond both ends of the phantom. The standard convolution kernel for chest was used (Br38) and a reconstructed slice thickness (ST) of 2 mm was selected. However, in the context of the investigation of the effects of different acquisition and reconstruction parameters, additional scans and reconstructions were made with Mercury 4.0 using: a) Pitch factors of 0.6, 1, and 1.2 (HF direction only), b) two additional clinical examination protocols for abdomen, one helical and one axial (both scanning directions). Details about the scan acquisitions are presented in Table 2. All scans were performed using a fixed kVp value of 120 kV.

**TABLE 2:** Description of the scan acquisitions and the reconstructed series presented in this study. In the last column the figures at which these data are shown are given. In bold are given the acquisitions that were performed with high dose examinations protocols

ID No	Set No	Acq. No	Series No	Phantom	Scan dir.	Protocol^a^	Convolution kernel (reconstructed ST)	Pitch	CTDI_vol_ (mGy)	DLP (mGycm)	Figure
1	A	5	9	Mercury	HF	Thorax Plain	Br38 (2 mm)	0.8	2.87	186.2	2,3,10
2	A	6	12	Mercury	FH	Thorax Plain	Br38 (2 mm)	0.8	2.83	183.5	2,3
3	A	3	3	Mercury	HF	Thorax Plain	Br38 (2 mm)	0.6	2.87	184.6	10
4	A	13	33	Mercury	HF	Thorax Plain	Br38 (2 mm)	1	2.9	189.8	10
5	A	14	36	Mercury	HF	Thorax Plain	Br38 (2 mm)	1.2	2.98	197.1	10
**6**	**B**	**38**	**4**	**Mercury**	**HF**	**Abdomen**	**Br38 (2** **mm)**	**0.8**	**6.79**	**457.0**	**9**
**7**	**B**	**39**	**5**	**Mercury**	**FH**	**Abdomen**	**Br38 (2** **mm)**	**0.8**	**6.71**	**451.3**	**9**
**8**	**B**	**20–37**	**3**	**Mercury**	**HF**	**AbdSeq^b^**	**Br38 (2** **mm)**	**0.9^a^**	**9.09**	**564.3**	**8**
**9**	**B**	**2–19**	**2**	**Mercury**	**FH**	**AbdSeq^b^**	**Br38 (2** **mm)**	**0.9^a^**	**9.09**	**564.3**	**8**
10	C	14	27	CTDI‐P1	HF	Thorax Plain	Br38 (2 mm)	0.8	2.11	92.18	4,6
11	C	15	30	CTDI‐P1	FH	Thorax Plain	Br38 (2 mm)	0.8	2.11	92.18	4,6
12	C	2	2	CTDI‐P2	HF	Thorax Plain	Br38 (2 mm)	0.8	2.36	103.26	5,6
13	C	3	5	CTDI‐P2	FH	Thorax Plain	Br38 (2 mm)	0.8	2.36	103.26	5,6
14	C	25	50	CTDI‐T	HF	Thorax Plain	Br38 (2 mm)	0.8	3.12	98.24	7
15	C	26	54	CTDI‐T	FH	Thorax Plain	Br38 (2 mm)	0.8	3.08	96.9	7

^a^ The reference mAs are: 66 for Thorax Plain and 210 for the Abdomen and AbdSeq.

^b^ Axial scan.

The imQuest v7.1 software (Clinical Imaging Physics Group, Department of Radiology, Duke Health, imquest@duke.edu) that has been produced for evaluation of image quality with the Mercury phantom (and the ACR 464 phantom) according to the methodology described in the AAPM TG233 report, was used in this study for producing: a) the water equivalent diameter (WED) of each phantom with respect to the Z‐axis value (table position), b) various image quality indices, including the detectability index (d’) which is the only image quality parameter that will be presented in this paper (applicable only to the Mercury phantom). A free software (ImageJ 1.53a, https://imagej.nih.gov/ij/index.html) and in‐house build macros were also used to read the CT number and standard deviation (SD) of the reconstructed CT images. A free software (DICOM Info Extractor^13^) was used to read the DICOM headers of the reconstructed CT images, to derive information about the scanning and reconstruction parameters of each image, including the table position values and the respective mA and CTDI_vol_ values.

For all phantoms, the ATCM modulation curves were created in terms of CTDI_vol_ versus table position of each reconstructed CT image (using the DICOM data), to present the dose modulation rather than mA modulation only, to also account for the effect of rotation time and pitch. For each one of these ATCM curves (henceforth referred to as dose modulation curves) two additional curves were constructed: WED (secondary Y‐axis) versus table position, and SD of each image (secondary Y‐axis) versus table position. All these curves were superimposed on scan projection radiograph images, where the positions of the first and the last image positions of each scan series are also depicted, so as to be able to observe the dose modulation (primary Y‐axis), with respect to the different phantom sections. In this way, the result of dose modulation was visually studied in terms of simultaneous variations of CTDI_vol_, WED and SD with respect to the position of the CT image within the phantom. The main results of the above analysis are also presented in additional graphs in terms of CTDI_vol_ versus WED and in Table 3, where the d’ values for the five rods from different materials contained within the five different diameter cylinders of the Mercury phantom are also given.

**TABLE 3:** Results of detectability values of the five different rod materials present the five different nominal WED equivalent diameters (values in parenthesis, in mm) of the Mercury phantom, obtained using the imQuest software

	Bone						Air						Iodine				
ID No	d' (160)	d' (210)	d' (260)	d' (310)	d' (360)		d' (160)	d' (210)	d' (260)	d' (310)	d' (360)		d' (160)	d' (210)	d' (260)	d' (310)	d' (360)
**1**	241.7	172.2	146.6	121.2	86.1		197.8	158.0	127.9	112.3	79.4		87.0	58.7	50.6	40.0	30.3
**2**	402.2	170.8	131.9	120.1	82.2		328.7	160.0	116.6	109.2	76.3		144.9	57.9	45.4	41.0	28.2
**3**	276.7	167.2	145.3	117.9	87.5		225.3	152.4	127.4	109.1	81.3		99.1	56.8	49.7	39.0	30.0
**4**	245.2	164.1	152.9	123.1	87.4		201.1	149.2	134.9	113.2	80.7		88.4	55.2	51.4	42.2	30.9
**5**	143.9	165.1	177.3	126.7	84.3		134.3	151.5	158.9	116.2	77.8		36.4	55.8	61.2	43.1	29.4
**6**	**412.1**	**282.8**	**259.7**	**205.6**	**160.8**		**334.2**	**239.8**	**226.8**	**185.5**	**148.1**		**148.0**	**90.4**	**89.1**	**69.0**	**55.6**
**7**	**628.2**	**287.5**	**244.5**	**201.0**	**154.7**		**500.5**	**249.1**	**217.9**	**186.0**	**143.6**		**220.1**	**101.0**	**84.1**	**69.3**	**52.9**
**8**	**484.2**	**214.9**	**174.7**	**145.8**	**110.5**		**404.1**	**187.0**	**157.9**	**135.9**	**105.7**		**171.1**	**69.3**	**58.7**	**47.2**	**37.5**
**9**	**385.4**	**215.3**	**181.8**	**149.8**	**119.3**		**306.5**	**191.6**	**164.2**	**141.7**	**113.0**		**130.9**	**74.0**	**60.8**	**49.9**	**40.2**

	Water						Polysterene										
ID No	d' (160)	d' (210)	d' (260)	d' (310)	d' (360)		d' (160)	d' (210)	d' (260)	d' (310)	d' (360)						
**1**	20.7	15.4	9.9	7.9	6.7		11.6	6.6	5.5	4.5	4.0						
**2**	35.5	14.4	9.3	8.3	6.0		17.4	6.4	5.1	4.2	4.0						
**3**	23.8	14.0	10.3	8.0	7.0		13.0	6.6	5.2	4.2	4.0						
**4**	21.0	13.8	11.5	8.6	6.8		10.8	6.7	4.5	4.7	3.2						
**5**	15.5	14.2	12.3	8.2	5.7		1.1	5.8	7.3	4.5	4.3						
**6**	**35.4**	**23.2**	**20.6**	**16.0**	**12.6**		**20.8**	**9.9**	**9.0**	**7.7**	**7.6**						
**7**	**53.5**	**23.3**	**19.5**	**15.6**	**12.6**		**32.2**	**12.5**	**10.2**	**8.6**	**6.5**						
**8**	**40.8**	**16.9**	**13.4**	**10.8**	**8.2**		**22.4**	**8.3**	**7.2**	**6.5**	**5.1**						
**9**	**31.7**	**16.5**	**12.4**	**10.6**	**9.5**		**17.8**	**8.7**	**7.7**	**6.3**	**5.4**						

More details about the acquisition parameters are given in Table 2. In bold are given the detectability values for the acquisitions that were performed with high dose examinations protocols.

## 3 RESULTS

**FIGURE 2:**
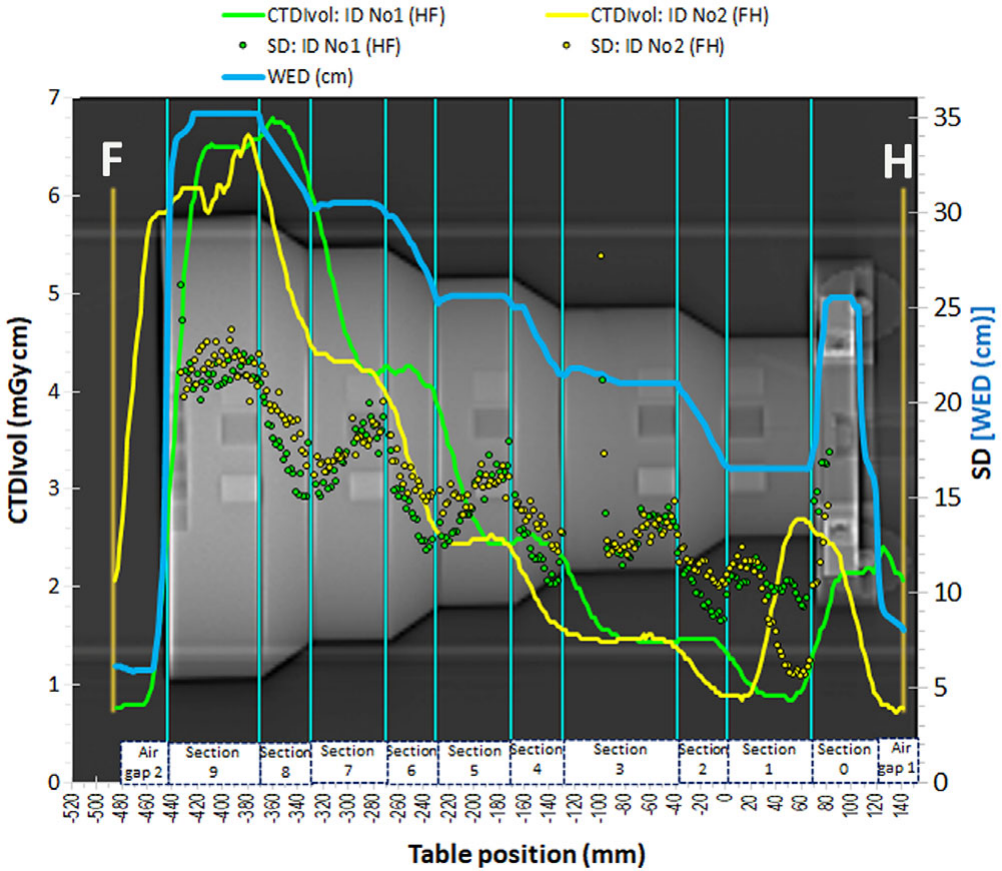
Dose modulation curves and respective SD and WED values for acquisitions ID No 1 (HF) and ID No 2 (FH). The letters H and F indicate the Head and Feet directions, respectively

The CTDI_vol_ modulation curves for the acquisitions ID No 1 (HF) and ID No 2 (FH) using the Mercury phantom are depicted in **Figure** **2**. Apparently, the two dose modulation curves differ much from each other, and neither of them follows closely the WED curve (secondary Y‐axis). The WED curve was produced by the imQuest software using the reconstructed CT images obtained from each scan series, and practically there was no difference between the WED curves produced for the HF and FH scan series.

When looking at both modulation curves starting from positive table position values (Head) and moving to negative table position values (Feet), it appears that the dose modulation curve for the HF scanning direction (HF scan) precedes the WED increments present in the WED curve along the HF direction. For example, the first CTDI_vol_ peak is observed in the Air gap 1 region, about 20 mm prior to the abrupt increase in WED observed in Section 0. On the other hand, the dose modulation curve for the FH scanning direction (FH scan) seems to present a considerable delay regarding the WED increase observed in Section 0, since the first CTDI_vol_ peak is found in Section 1. The difference between these two CTDI_vol_ peaks is about 60 mm. When moving towards the other sections of the Mercury phantom in the HF direction, it can be seen that the spatial difference between the two dose modulation curves varies and decrease to about 40 mm, while at some points along the phantom the two curves intersect. The dose modulation curve (HF scan) starts to move abruptly downwards about 30 mm before the end of the thickest section of the phantom (Section 9), whereas the dose modulation curve (FH scan) starts descending abruptly about 20 mm after the end of the phantom, without reaching the minimum CTDI_vol_ of the dose modulation curve (HF scan) observed in the Air gap 2 region. Despite the differences observed between the dose modulation curves for the two different scanning directions, the CTDI_vol_ and DLP values for the two respective acquisitions were practically the same, as can be seen in Table 2.

**FIGURE 3:**
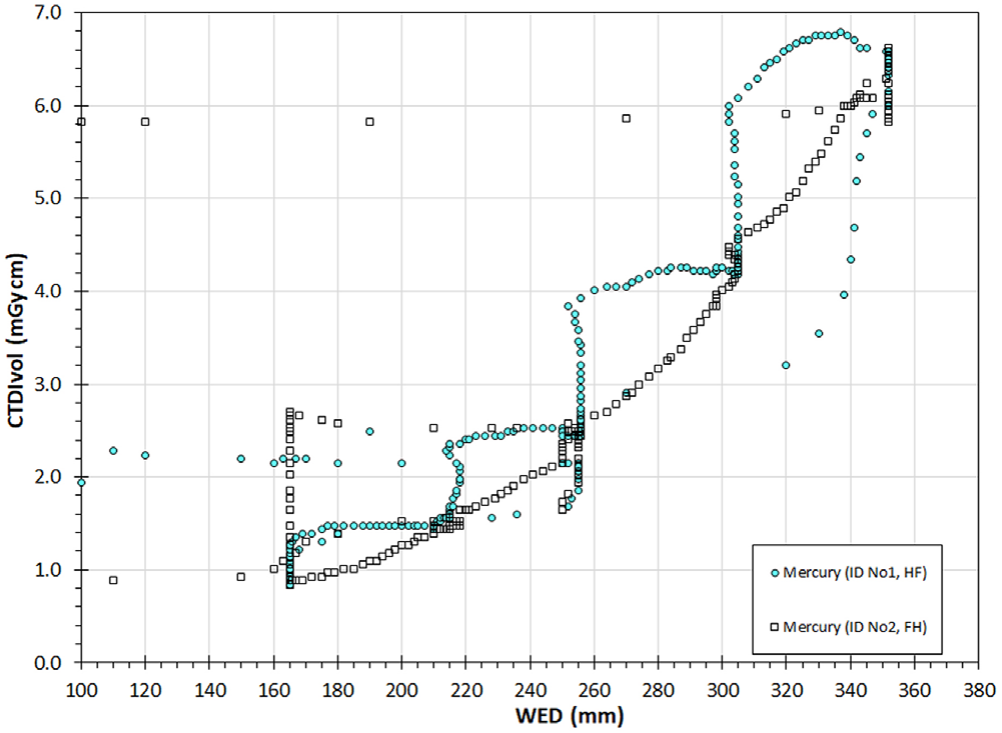
The CTDI_vol_ values of Figure 2 are given in relation to the respective WED values calculated by the imQuest software

However, when considering that the dose modulation curve (FH scan) results when the Mercury phantom is scanned in the opposite direction than the dose modulation curve (HF scan), it becomes rather clear that the changes in the dose modulation curve for the FH scanning direction variations also precede the respective WED variations. A closer look at the modulation curves reveals that the dose modulation curve (FH scan) follows closer to the WED variations, as the CTDI_vol_ decreases with WED decrease at the cone rings (Sections 8, 6, 4, and 2) and remains relatively constant at the cylindrical regions (Sections 7, 5, and 3). On the contrary, the dose modulation curve (HF scan) exhibits a mixed behavior, for both cylindrical and conical parts of the phantom. For example, the CTDI_vol_ is relatively constant in Section 2 (cone ring) instead of increasing, it increases in Sections 5 and 7 (cylinders) instead of remaining constant, and it increases in Section 8 (cone ring) as it should. This behavior can be better appreciated in Figure 3 where the CTDI_vol_ data points of Figure 2 are given in relation to the respective WED value (in mm) of the phantom at each table position. Except for some outliers (reflecting mostly data points near transition zones), it can be seen in Figure 3 that the CTDI_vol_ values of the dose modulation curve (FH scan) follow an exponential‐like variation in response to the respective WED variations, in contrast to the dose modulation curve (HF scan) which looks more like a four‐step function.

In Figure 2 are also depicted the SD values of the respective CT images acquired from the HF and FH scans. To obtain the SD values, a region of interest (ROI) with the shape of a cylindrical band was positioned around the central axis of the phantom. It must be noted that the SD values were considered valid only for those CT images where the mean CT# of the ROI was within the range of HU values expected for the phantom material (−110 to −70 HU) to avoid considering the SD values for CT images positioned in transitional regions between slices, within the solid water ramp (it is located in Section 1 and is used for Z‐axis resolution measurements) or the air gap regions. Despite the fluctuations observed, it can be seen that the SD values which correspond to the dose modulation curve for the FH scanning direction were in general larger than the respective SD values for the dose modulation curve for the HF scanning direction. An exception exists in Section 1, where the ‘delayed’ increase of the CTDI_vol_ values observed in the dose modulation curve (FH scan), resulted in smaller SD values. The variation of SD values with WED depicted in Figure 2 also indicates that the SD values increase with increasing WED and therefore the dose modulation does not fully compensate for thickness variations, so as to maintain the noise levels constant.

**FIGURE 4:**
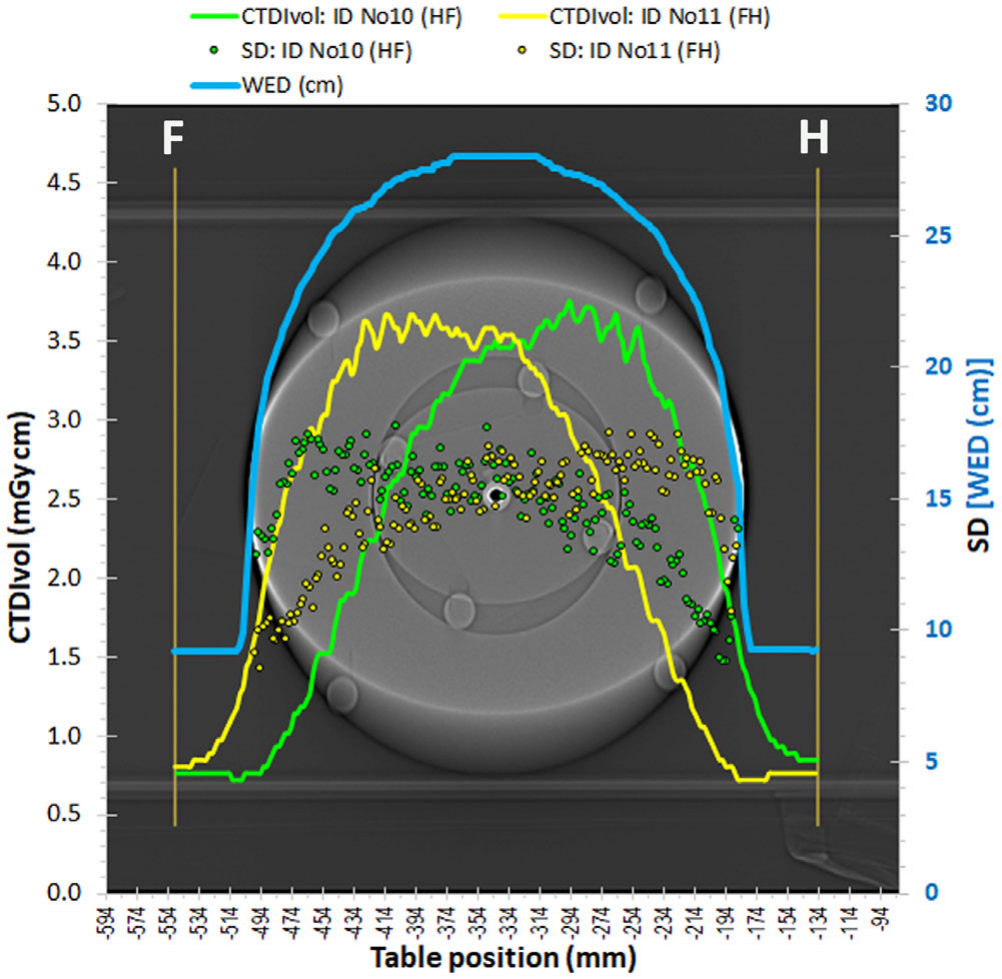
Dose modulation curves for acquisitions ID No 10 (HF) and ID No 11 (FH) and respective SD values with the phantom CTDI‐P1

The dose modulation curves derived using the CTDI‐P1 phantom and opposite scanning directions are depicted in Figure 4. It can be seen that for the two opposite scanning directions large differences between the two dose modulation curves were observed, as with the Mercury 4.0 phantom. Despite these differences, the CTDI_vol_ and DLP values for the two respective acquisitions were practically the same (Table 2). The two dose modulation curves are not symmetrical around the phantom center, as someone would expect based on the symmetry of the CTDI‐P1 phantom. On the contrary, they look like mirror images around the phantom's center. When looking at the dose modulation curve (HF scan) in the HF direction, it is evident that the CTDI_vol_ values start rising before even reaching the phantom (within the air gap region), peak before the phantom middle and decrease thereafter, reaching the minimum slightly before the end of the phantom. When looking at the other dose modulation curve (FH scan) in the FH direction, the same observations apply.

**FIGURE 5:**
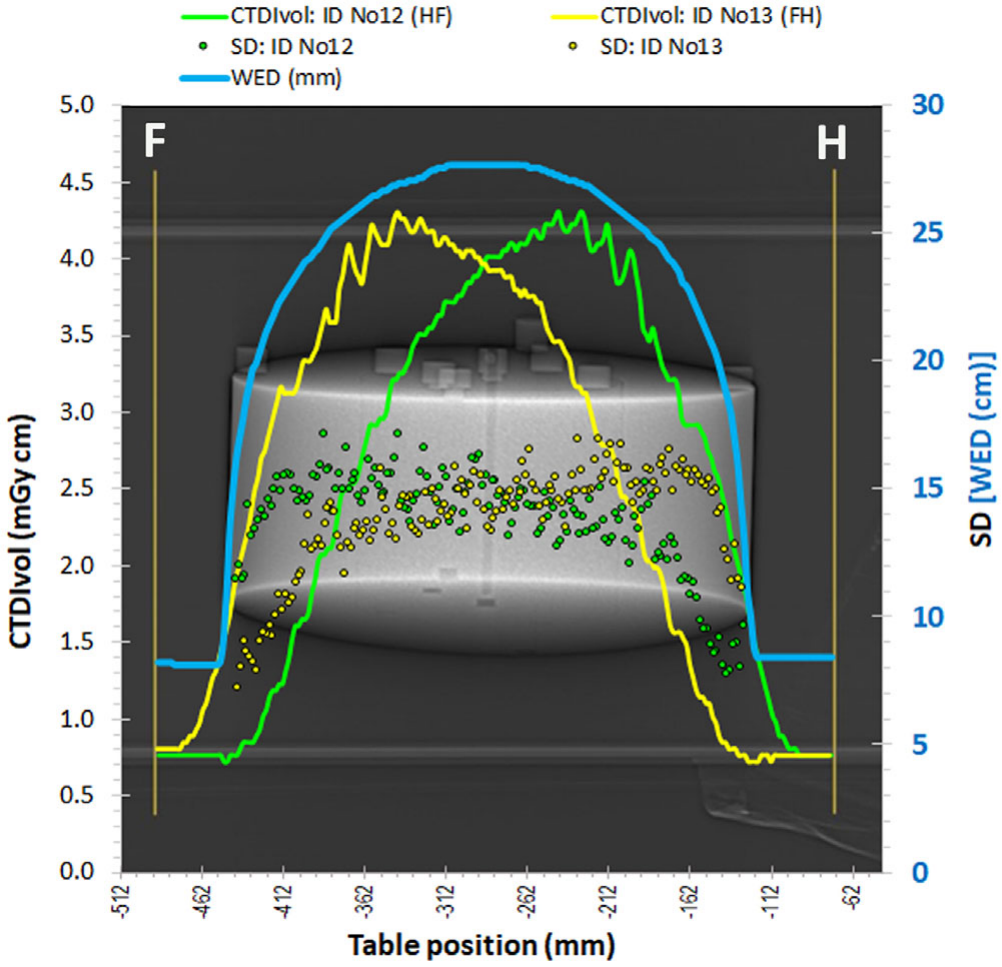
Dose modulation curves for acquisitions ID No 12 (HF) and ID No 13 (FH) and respective SD values with the phantom CTDI‐P2

The dose modulation curves derived using the CTDI‐P2 phantom are depicted in Figure 5. As with the CTDI‐P1, the dose modulation curves for different scanning directions were different and the same observations as for Figure 4 apply, regarding their shape. The CTDI_vol_ and DLP values for the two respective acquisitions with CTDI‐P2 were the same (see Table 2), but the CTDI_vol_ values in Figure 5 were larger compared to the respective values observed in Figure 4, though the WED curves were practically the same. This could be attributed to the fact that the ATCM systems increase the mA when the larger thickness is observed in the AP direction (*Y*‐axis) rather than the LAT direction (*X*‐axis).^3^ Regarding the SD values also depicted in Figure 5, it can be seen again that the asymmetry in the dose modulation curves resulted in a reversed asymmetry in the SD values. The same methodology was used to derive the SD values, as with the CTDI‐P1 phantom.

**FIGURE 6:**
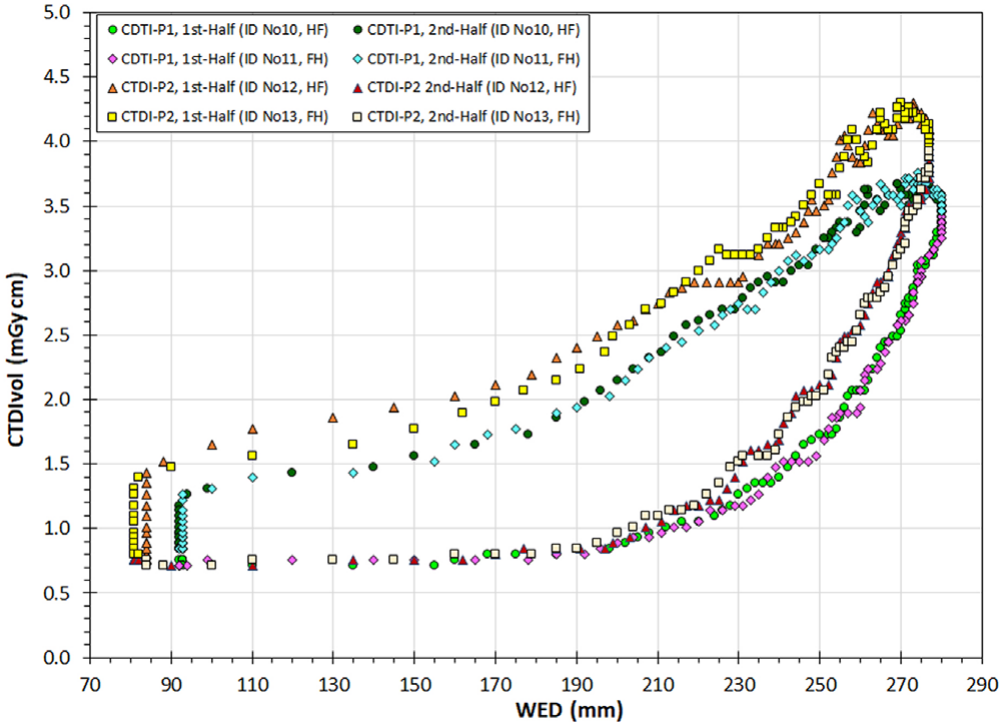
The CTDI_vol_ values of Figures 4 and 5 are given in relation to the respective WED values calculated by the imQuest software

In Figure 6, the CTDI_vol_ values of Figures 4 and 5 are given in relation the WED values (in mm) of the CTDI‐P1 and CTDI‐P2 phantoms at each table position. With the exception of some data points around 80 mm (points near phantom‐air transition zones), this graph proves the fact that for both scanning directions, the CTDI_vol_ values are larger when scanning the first half of the phantom, compared to the respective values arising when scanning the second half, which has been recently reported in terms of mA modulation by Papadakis and Damilakis.^11^

**FIGURE 7:**
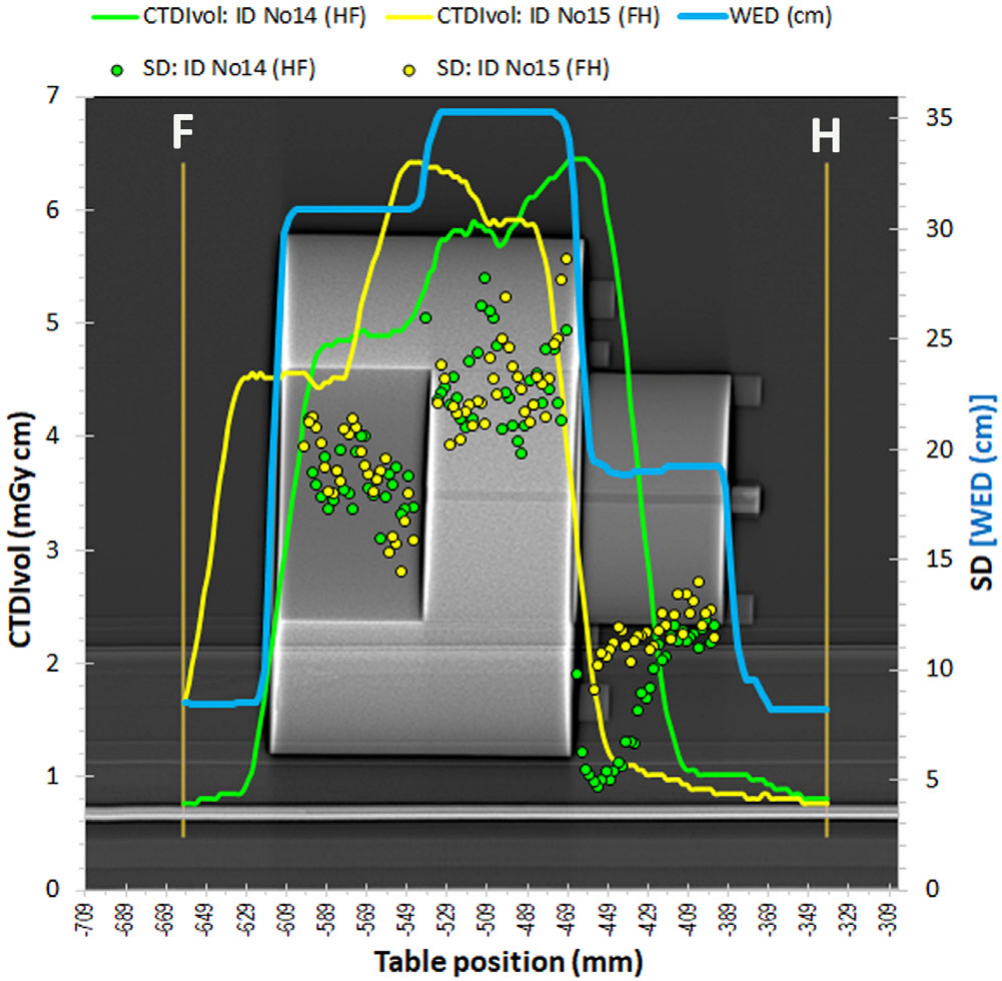
Dose modulation curves for acquisitions ID No 14 (HF) and ID No 15 (FH) and respective SD values with the phantom CTDI‐T

In Figure 7 are shown the dose modulation curves and the resulting SD values with the CTDI‐T phantom (3‐section phantom: head, abdomen, chest) for the HF and FH scanning directions. As with the Mercury 4.0 phantom and the CTDI‐P1 and CTDI‐P2 phantoms, the dose modulation curves for the HF and the FH scanning directions, and the respective SD curves, presented large differences, though the CTDI_vol_ and DLP values for both scans were practically the same (see Table 2). Regarding the SD values, it should be noted that two ROIs were used to monitor the SD values: one in the head phantom area (head ROI) and one in the body ring area (body ROI), since in the lung section the head ROI is within the air and in the head section the body ROI is in the air. To derive the SD values, the same rules were applied as with the CTDI‐P1 and CTDI‐P2 phantoms.

**FIGURE 8:**
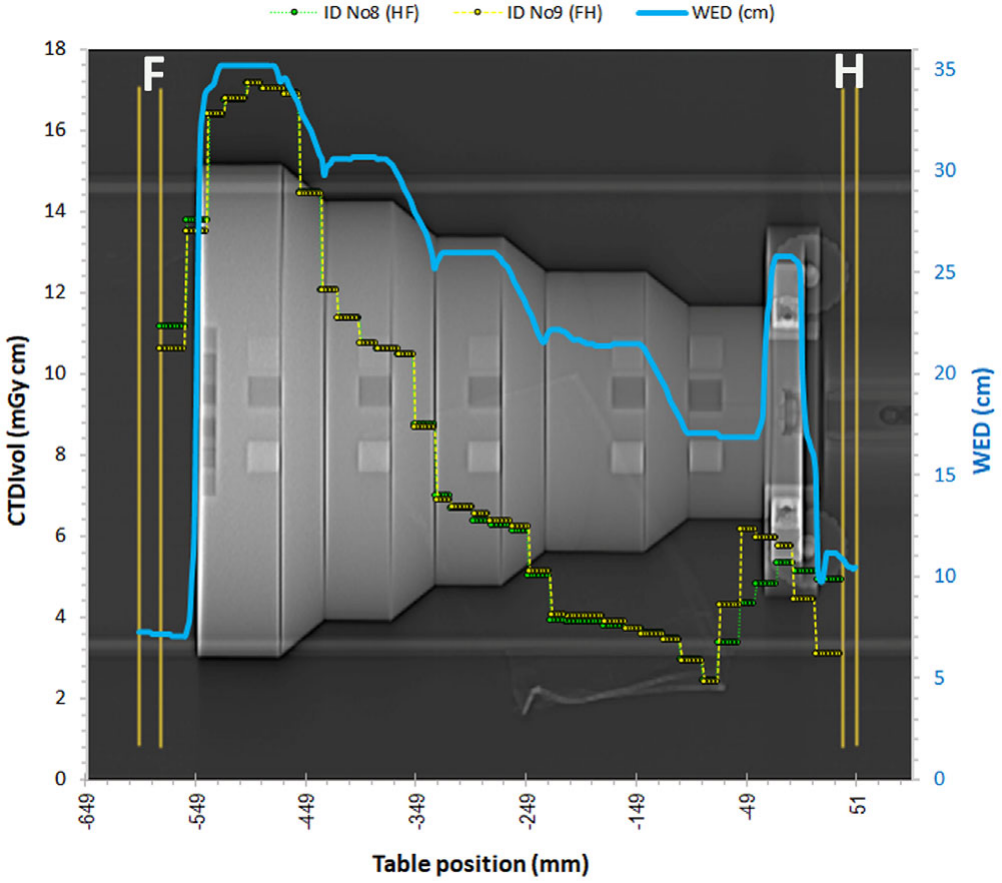
Dose modulation curves for axial acquisitions ID No 8 (HF) and ID No 9 (FH) with the Mercury phantom. The vertical orange lines indicate the planed scanned length of the helical (outer lines) and axial (inner lines) acquisitions

The response of the ATCM system dose at the interfaces may be better comprehended by looking at the dose modulation curves for the axial acquisitions (ID No 8 and 9), shown in Figure 8. The scalar variation and the position of peaks suggest that the modulation is affected by the thickness of the material that lays ahead of the table position of each image slice in the scanning direction, but it is not only the maximum WED value that determines the CTDI_vol_ value. Since the X‐ray beam in all these acquisitions had a finite width of 38.4 mm, it should be expected that mA change in steps equal to the beam width (in line with what was mentioned when commenting on Figure 6). However, since the tube mA changes during tube rotation (angular modulation) to account for differences in thickness in AP and LAT directions (as has been seen in Figures 4 and 5 and reported elsewhere^1^, ^2^, ^3^, ^10^), the modulation can be observed in smaller steps. Indeed, it was observed that for the axial acquisition in the HF direction (ID No 8) the CTDI_vol_ changes in steps of 14, 16, 20, 24 and 34 mm, whereas for the axial acquisition performed in the FH direction (ID No 9), in steps of 16, 20 and 24 mm.

To understand how this modulation works, it was attempted to find a relationship that fits the CTDI_vol_ values of the dose modulation curves of the helical acquisitions considering the respective WED curves. Several relationships were tested and the one that gave the best fit was the following:

(1) $$CTDI_{vol}=\;a\times \;\left[\exp\left(f{\left(WED\right)}_{z+d}\;\times b\right)\right]+c$$

(2) $$\begin{array}{ccc} & & f{\left(WED\right)}_{z+d}=w_{1}\;\times \mathrm{maximum}{\left(WED\right)}_{z+d}+w_{2}\\ & & \;\times \;3\mathrm{rdQuartile}{\left(WED\right)}_{z+d}+w_{3}\times \mathrm{Median}{\left(WED\right)}_{z+d}\\ & & \;+\;w_{4}\;\times \;\mathrm{minimum}{\left(WED\right)}_{z+d} \end{array}$$

where a,b,c,d,w_1_,w_2_,w_3_,w_4_ are fitting parameters (w_1_+w_2_+w_3_+w_4 _= 1), z is the table position of each reconstructed slice and d is the range of images ahead that is taken into account in the CTDI_vol_ (i.e., the mA) value selection.

**FIGURE 9:**
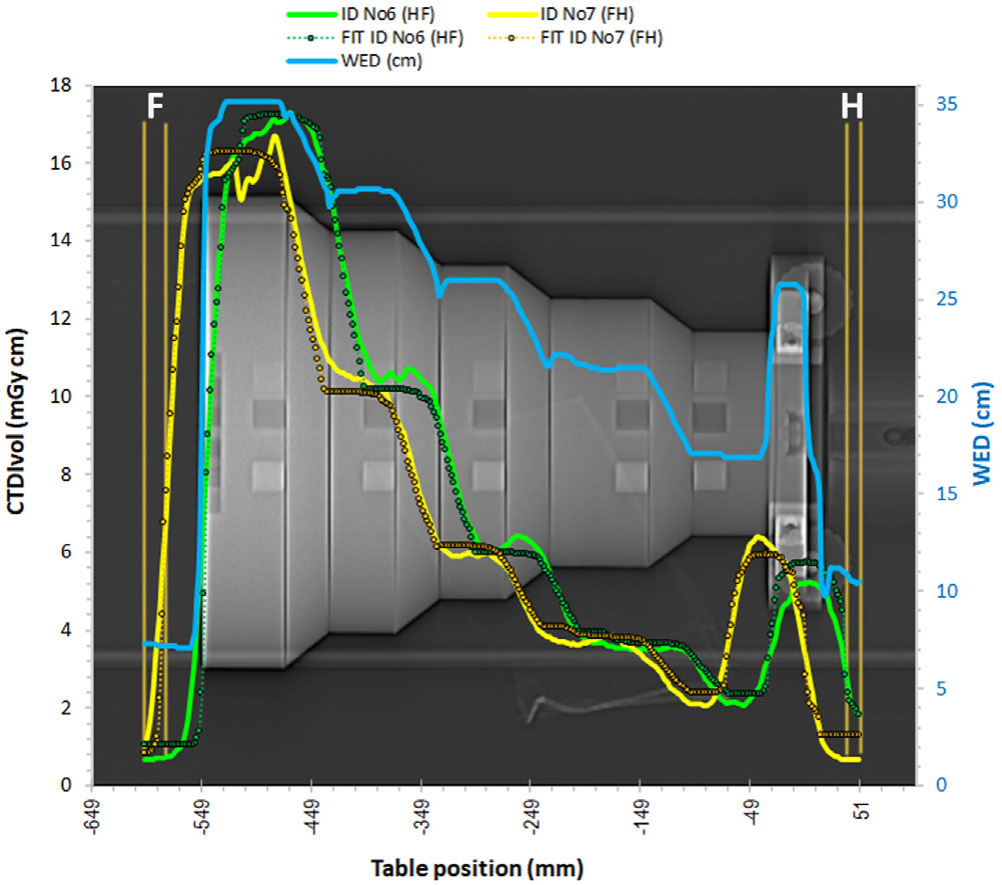
Dose modulation curves for acquisitions ID No 6 (HF) and ID No 7 (FH) with the Mercury phantom and respective fitted curves. The vertical orange lines indicate the planed scanned length of the helical (outer lines) and axial (inner lines) acquisitions

Equation (1) is the same as Equation (3) proposed by Ria et al.^14^ for fitting the CTDI_vol_ data from the Mercury phantom using the WED diameters of the five cylinders and comparing them with data from dose modulation curves in actual patients. The difference is that combining Equation (1) with Equation (2) we attempted to document that for the mA modulation it is not only the maximum WED value that is considered. The fitting results were not perfect as expected, since the resulting CTDI_vol_ of each slice is just an average of the values that correspond to the whole rotation. However, it was deduced that the WED values of about 20 slices (40 mm) ahead of the table position that correspond to each CT image towards the scanning direction (HF or FH) are considered for the mA modulation of the scans derived with pitch 0.8, and it is not only the maximum and minimum WED value within this table positions’ range that is considered, since the best fitting was obtained with none of the w_1_,w_2_,w_3_ and w_4_ being zero (a single set of fitting parameters was used for both modulation curves). The results of this fitting method for the acquisitions performed with the Mercury 4.0 phantom and the abdomen protocol (ID No 6 and 7) are shown in Figure 9. Similar agreement between the actual and the fitted dose modulation curves was observed for the data derived using the three CTDI phantom configurations.

**FIGURE 10:**
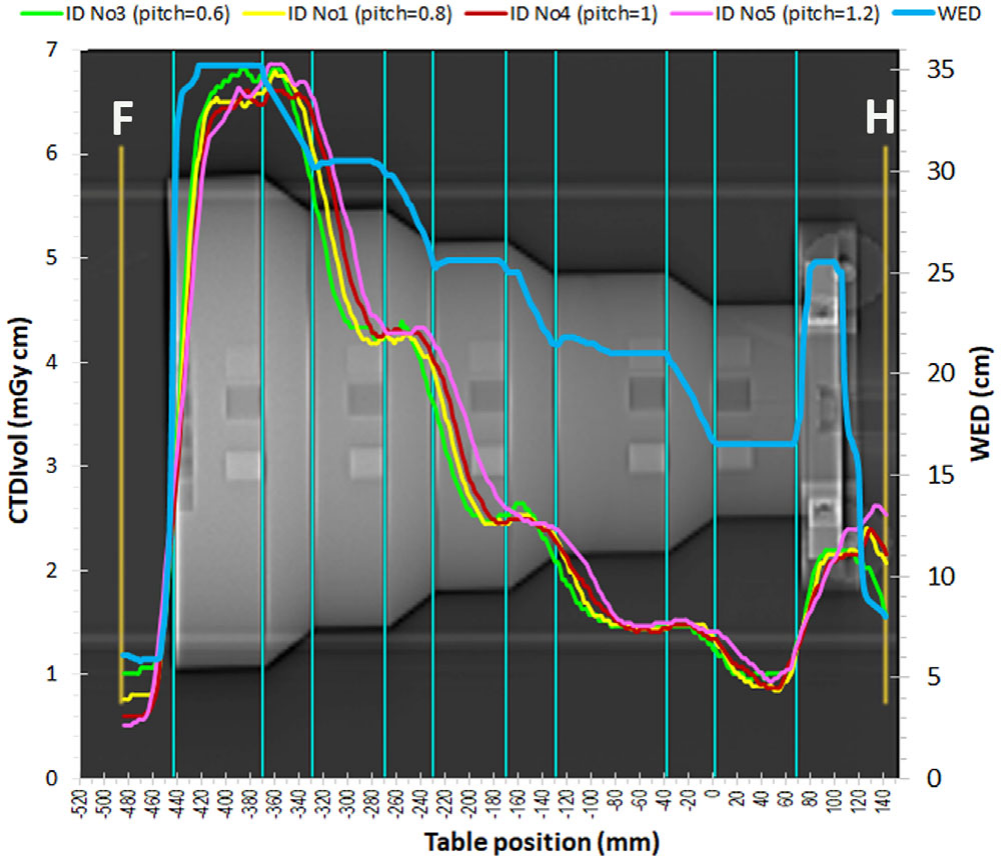
Dose modulation curves for acquisitions ID No 3 (pitch = 0.6), ID No 1 (pitch = 0.8), ID No 4 (pitch = 1.0) and ID No 5 (pitch = 1.2) with the Mercury phantom (all HF)

The dose modulation curves for acquisitions ID No 3 (pitch = 0.6), ID No 1 (pitch = 0.8), ID No 4 (pitch = 1.0) and ID No 5 (pitch = 1.2) with the Mercury 4.0 phantom are shown in Figure 10. It appears that when scanning is made using a larger helical pitch, the ability of the ATCM system to follow the WED changes along the z‐axis is impaired, because with a large pitch the effective X‐ray beam width in relation to ATCM is increased.^10^, ^11^ Indeed, the best fitting results using Equations (1) and (2), were obtained using d values of 16 (32 mm), 20 (40 mm), 25 (50 mm) and 26 (52 mm) for pitch values of 0.6, 0.8, 1, and 1.2, respectively (all the rest fitting parameters were identical).

**FIGURE 11:**
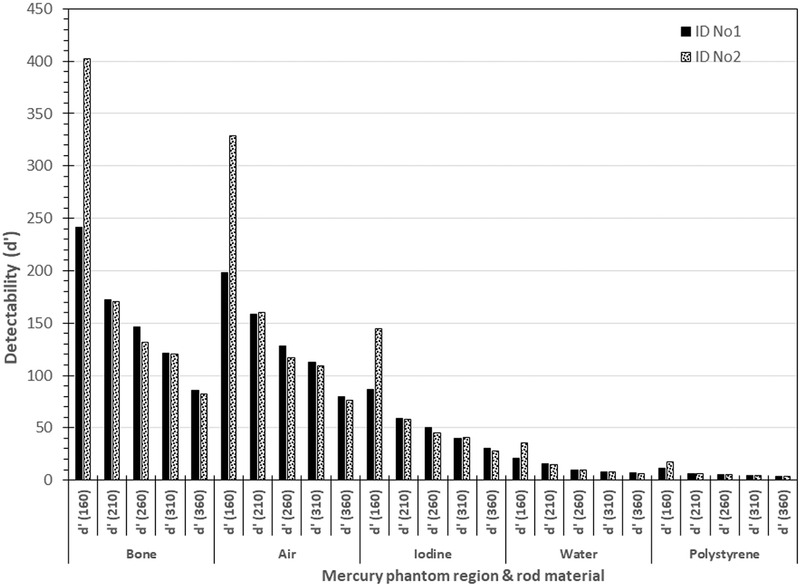
Comparison of detectability (d’) values for acquisitions ID No 1 (HF) and ID No 2 (FH) with the Mercury phantom

Finally, results of the image quality evaluations of the acquisitions of Table 2 derived using the imQuest software package (for the Mercury phantom only) are summarized in Table 3. It can be seen that the detectability (d’) values for bone are the highest, followed by those for air, iodine, water and polystyrene. Furthermore, it was observed that for all materials the d’ values are larger for the smaller nominal WED diameters of the Mercury phantom and for the high dose protocols (Abdomen and AbdSeq) with respect to the ThoraxPlain protocol acquisitions. In Figure 11 a graphical comparison of detectability (d’) values is presented for the acquisitions made using the HF (ID No 1) and the FH (ID No 2) scanning directions. It can be seen that the d’ values increase for the phantom regions where the CTDI_vol_ values were larger, leading to either smaller noise values or/and larger Task Transfer Function (TTF) values, since d’ is a function of TTF and noise power spectrum (NPS), and increases for larger TTF values and smaller values of noise.

## 4 DISCUSSION

According to the results presented the main findings of this study are the following:

- The scanning direction greatly affects the modulation curves, since from the fitting procedure it is suggested that at each table position the WED values of the phantom structures that lay ahead (towards the scanning direction) within the X‐ray beam width range, are accounted to select the right tube current and consequently the right dose. The effect of the scanning direction on dose modulation was verified using all four phantoms. Therefore, even when scanning a perfectly symmetrical phantom, like the CTDI‐P1 and CTDI‐P2 the dose modulation curves for opposite scanning directions may differ in other CT scanners as well.^11^

Despite what suggested by the results of the fitting procedure, it was notable that a unique set of fitting parameters that could reproduce the dose modulation curves for all the phantoms could not be found. The exact way that the mA values are calculated is not known but most probably it is based on a proprietary algorithm, that may use the WED values (or pixel values) of the SPR images, to determine which is the most attenuating phantom segment that is included within the X‐ray beam width, so as to adjust the mA for the maximum WED and also apply angular modulation at different rotation angles.^1^, ^2^ It is also possible for the algorithm to use WED thresholds, to increase or decrease the strength of modulation depending on the examination protocol (reference mAs), and the ATCM adjustment. However, it was difficult to understand how any of these features could justify the effect of the scanning direction. A mathematical model that was built to study this effect suggested that the explanation is rather complex and requires further investigation which goes beyond the scope of this paper.

- The current adjustment of ATCM modulation strength does not fully compensate for the larger phantom thickness, since the phantom regions with larger WED present larger noise. This was also verified using all four phantoms.
- The dose modulation is affected by the pitch selection, and with smaller pitch values the ability of the ATCM system to follow the WED changes along the z‐axis is improved, since the effective X‐ray beam width is reduced.
- Apart from WED, the dose modulation is greatly affected by the noise level required for each examination protocol. As can be seen in Table 3, the d’ values for the abdomen helical and axial acquisitions (ID No 5–9) are much larger than those obtained with the Thorax protocol. The only exception is the smallest WED diameter, where the d’ values for the acquisition ID No 2 are comparable or even larger than the respective values of the acquisitions ID 6, 8 and 9, due to the large CTDI_vol_ values at the uniform part of the smallest diameter cylinder where the noise is determined. It must be noted that as can be seen in Figures 2, 8, 9, and 10, for acquisitions made with ATCM, the mA will usually vary within each phantom section. Therefore, the d’ values which are obtained as a function of TTF and noise properties in phantom sections scanned with different mA values, may be inaccurate. However, the investigation of this problem and other aspects of image quality evaluation with the imQuest software is beyond the scope of this paper. The superior image quality of the Abdominal protocols was expected considering that the reference mAs for the Abdominal protocols was set at 210 mAs, whereas for the Thorax protocols was set to 66 mAs, and the average CTDI_vol_ values of the helical abdominal and axial abdominal scans were respectively more than twice and three times the CTDI_vol_ value of the thorax protocol.

The variation of SD values with WED depicted in Figures 2, 4, 5, and 7 and the different d’ values observed in phantom sections with different WED shown in Table 3, clearly indicate that while according to the basic principle of AEC systems the dose modulation aims to compensate for thickness increases to keep the noise levels constant, this goal is not accomplished, since the SD values increase and the d’ values decrease with increasing WED. However, this does not mean that the AEC does not operate properly. Indeed, Siemens CARE Dose 4D ATCM system has a variable level of tube current adjustment for compensating for thickness variations.^1^, ^11^ "Weak," "average," or "strong" compensation settings can be used to provide a relatively low, medium or high degree of mA adjustment, separately for patients which are larger or smaller than the reference patient. (The default modulation strength setting in CARE 4D dose ATCM system is average decrease for slim patients and average increase for obese patients^11^). All three settings result in less tube current adjustment than this that would be necessary to keep image noise constant for all patient sizes.^1^ This approach is supposed to match clinical requirements more closely, since smaller patients require smoother images as it is more difficult to differentiate organs correctly when less fat is present.^1^ Other CT scanner manufacturers use different approach than Siemens and limit the minimum and maximum mA values that can be used by the ATCM system, to prevent extreme mA reductions in slim patients and extreme mA escalations in obese patients, respectively.^1^

It must be noted that for all phantoms, the air gaps before and after the phantom were intentionally used, to investigate how the ATCM responds in sudden changes, that is from air to a medium and from a medium to air. Apart from this, air gaps are also useful for detecting the min–mA allowed.^4^ Additional scans which were performed with the same exposure factors but with a scan length limited within the phantom boundaries (not described in the Materials and Method section nor included in Tables 2 and 3, (see Supporting Information file) gave dose modulations curves similar to those with air gaps except minor differences.

The main limitation of this study is that the results were obtained from a single modern CT scanner and therefore cannot be generalized to other CT scanners of the same or other manufacturers, though it is reasonable to assume that a similar behavior should be expected. A second limitation is that there are many additional parameters that are expected to affect dose modulation and the resulting image quality (including reconstruction thickness, single versus dual SPR, phantom centering, convolution kernel, iterative reconstruction method and strength etc.) which could not be presented in this paper because of space limitations. However, this study highlighted that the scanning direction is an important parameter which should be considered when the ATCM operational parameters of other CT scanners are explored, and also have set a framework for the measurements that should be performed when comparing different CT scanners and examination protocols.

It must be noted that the Mercury phantom is a better fit to the modern methods required to evaluate image quality in CT scanners using iterative reconstruction algorithms, since they are nonlinear and the conventional image quality metrics like the SD are of limited utility.^15^ A detailed description of the methods used to evaluate image quality in those systems using the Mercury phantom and the imQuest software can be found elsewhere.^14^ The CTDI‐P1 and CTDI‐P2 configurations cannot be used with the imQuest software to obtain d’ values due to the lack of rod structures. However, the CTDI‐T configuration could be used to obtain d’ values for the air, by removing two PMMA pins, one from the periphery or the center of the head phantom part, and one pin from the body ring. For example, for the HF acquisition with the CTDI‐T phantom (ID No 14), the d’ values for the central air hole in the head and abdomen sections were 177 and 68, respectively. Though this prospect seems feasible, further investigation and validation will be required.

## 5 CONCLUSION

The Mercury 4.0 phantom (in conjunction with the ImQuest software), is a dedicated phantom for a complete and in‐depth evaluation of the ATCM operation and the resulting image quality of modern CT scanners, using the new methodologies proposed in the AAPM TG233 report. However, in its absence, the CTDI configurations can be used instead, to investigate and decode the basic operation principles of the ATCM systems of CT scanners from different manufacturers, especially when the imQuest software is available to facilitate the WED calculations. If the imQuest software is not available, there are other free software (ImageJ and DICOM Info Extractor) which can be used instead to obtain the SD value curves as a function of dose modulation curves.

For the CT scanner tested, it was seen with all four phantoms that the operation of ATCM system is intensely affected by the scanning direction and that image quality is reduced with increasing phantom thickness. From acquisitions performed in the Mercury 4.0 phantom only, it was seen that the dose modulation and the resulting image quality are also affected by the pitch selection and the examination protocol selection.

## AUTHOR CONTRIBUTIONS

Ioannis A. Tsalafoutas and Shady AlKhazzam conceived the idea, designed and performed the data acquisition and analysis, and wrote the manuscript. Huda AlNaemi and Mohammed Hassan Kharita contributed to interpretation of data, revising and approval of the manuscript's final version.

## CONFLICT OF INTEREST

The authors have no conflict of interest to declare.

## Supporting information



Table A1

Figure A1

Figure A2

Figure A3

Figure A4

Figure A5

Figure A6

Figure A7
