# Supplementary material for: Evaluation of automatic tube current modulation of CT scanners using a dedicated and the CTDI dosimetry phantoms
Source: J Appl Clin Med Phys. 2022 Jun 9;23(7):e13620. doi: 10.1002/acm2.13620 (PMC9278667; doi:10.1002/acm2.13620)
Supplement: Supplementary file 1 — Table A1 [file ACM2-23-e13620-s003.docx]

**Evaluation of automatic tube current modulation of CT scanners**

**using a dedicated and the CTDI dosimetry phantoms**

- **Supplemental materials file -**

| **Table A1.** Description of the scan acquisitions and the reconstructed series presented in this supplementary material file. All acquisitions were performed the with the Thorax Plain examination protocol, reconstruction kernel Br38 and 120 kV. ID numbers starting with S, correspond to the acquisitions presented only in the supplementary material file (the rest are also presented in the manuscript). In the last column are given the figures at which these data are shown. | | | | | | | | | | |
| --- | --- | --- | --- | --- | --- | --- | --- | --- | --- | --- |
| ID No | Set No | Acq. No | Series No | Phantom | Scan dir. | Reconstructed  ST (mm) | Pitch | CTDI_vol_ (mGy) | DLP (mGycm) | Figure |
| 1 | A | 5 | 9 | Mercury | HF | 2 | 0.8 | 2.87 | 186.2 | A1, A2, A3 |
| 2 | A | 6 | 12 |  | FH | 2 | 0.8 | 2.83 | 183.5 | A1 |
| S1 | A | 15 | 39 |  | HF | 2 | 0.8 | 3.21 | 168.4 | A1 |
| S2 | A | 16 | 42 |  | FH | 2 | 0.8 | 3.16 | 167.2 | A1 |
| S3 | A | 5 | 10 |  | HF | 3 | 0.8 | 2.87 | 186.2 | A2 |
| S4 | A | 5 | 11 |  | HF | 5 | 0.8 | 2.87 | 186.2 | A2 |
| 3 | A | 3 | 3 |  | HF | 2 | 0.6 | 2.87 | 184.6 | A3 |
| 4 | A | 13 | 33 |  | HF | 2 | 1 | 2.9 | 189.8 | A3 |
| 5 | A | 14 | 36 |  | HF | 2 | 1.2 | 2.98 | 197.1 | A3 |
| S5 | A | 20 | 50 | CTDI-P1 | HF | 2 | 0.8 | 2.15 | 90.3 | A4, A5 |
| S6 | A | 19 | 47 |  | FH | 2 | 0.8 | 2.1 | 88.5 | A4, A5 |
| S7 | A | 22 | 56 |  | HF | 2 | 0.8 | 2.41 | 80.9 | A4 |
| S8 | A | 21 | 53 |  | FH | 2 | 0.8 | 2.41 | 80.9 | A4 |
| S9 | A | 19 | 49 |  | HF | 5 | 0.8 | 2.15 | 90.3 | A5 |
| S10 | A | 20 | 52 |  | FH | 5 | 0.8 | 2.1 | 88.5 | A5 |
| 12 | A | 2 | 3 | CTDI-P2 | HF | 2 | 0.8 | 2.36 | 103.26 | A6 |
| 13 | A | 2 | 5 |  | FH | 2 | 0.8 | 2.36 | 103.26 | A6 |
| S11 | A | 9 | 21 | CTDI-T | HF | 2 | 0.8 | 3.12 | 98.2 | A7 |
| S12 | A | 10 | 24 |  | FH | 2 | 0.8 | 3.08 | 96.9 | A7 |
| S13 | A | 11 | 27 |  | HF | 2 | 0.8 | 3.84 | 90.6 | A7 |
| S14 | A | 12 | 30 |  | FH | 2 | 0.8 | 3.71 | 87.6 | A7 |

## **FIGURE LEGENDS**

**Figure A1.** Tube current (mA) modulation curves with the Mercury phantom, for acquisitions ID No1 (HF) and ID No2 (FH) with air gap and acquisitions ID NoS1 (HF) and ID NoS2 (FH) without air gap. The curves without air gap are similar to those with air gap, apart from some differences that appear mainly at the ends of the scans.

**Figure A2.** Tube current modulation (mA) curves and the respective SD values with the Mercury phantom, for acquisitions ID No1 (HF) with ST=2mm, ID NoS3 (HF) with ST=3mm and ID NoS4 (HF) with ST=5mm. The mA modulation curves are practically identical, whereas the SD values and slice-to-slice SD variations decrease with increasing slice thickness.

**Figure A3.** Tube current (mA) modulation curves for acquisitions ID No3 (pitch=0.6), ID No1 (pitch=0.8), ID No4 (pitch=1.0) and ID No5 (pitch=1.2) with the Mercury phantom (all HF). The large differences in mA presented in this graph are alleviated when the ATCM modulation curves are presented in terms of CTDI_vol_ (see Figure 10 in the manuscript).

**Figure A4.** Tube current (mA) modulation curves with the CTDI-P1 phantom, for acquisitions ID NoS5 (HF) and ID NoS6 (FH) with air gap, and acquisitions ID NoS7 (HF) and ID NoS8 (FH) without air gap, apart from some minor differences. The increase of mA at the end of the ID NoS6 curve is inexplicable.

**Figure A5.** Tube current (mA) modulation curves and respective SD curves with the CTDI-P1 phantom, for acquisitions ID NoS9 and ID NoS10 (HF) (ST=2 and 5mm, respectively), and for acquisitions ID NoS11 and ID NoS12 (HF) (ST=2 and 5mm, respectively). The increase of mA at the end of the ID NoS6 curve (two is inexplicable. It can be seen that, when ST is increased from 2 to 5 mm, SD is greatly reduced and slice to slice SD variations are also reduced, but do not disappear.

**Figure A6.** Dose modulation curves with the CTDI-P2 phantom, for acquisitions ID No12 (HF) and ID No13 (FH) are given, along with the respective fitted curves using equations 1 and 2 given in the main text. A single set of fitting parameters was used for both dose modulation curves.

**Figure A7.** Tube current (mA) modulation curves with the CTDI-T phantom, for acquisitions ID NoS10 (HF) and ID NoS11 (FH) with air gap, and acquisitions ID NoS12 (HF) and ID NoS13 (FH) without air gap. The curves without air gap are similar to those with air gap, apart from some differences observed mainly at the edges of the scans.
