# Supplementary figures and images for: Evaluation of automatic tube current modulation of CT scanners using a dedicated and the CTDI dosimetry phantoms
Source: J Appl Clin Med Phys. 2022 Jun 9;23(7):e13620. doi: 10.1002/acm2.13620 (PMC9278667; doi:10.1002/acm2.13620)

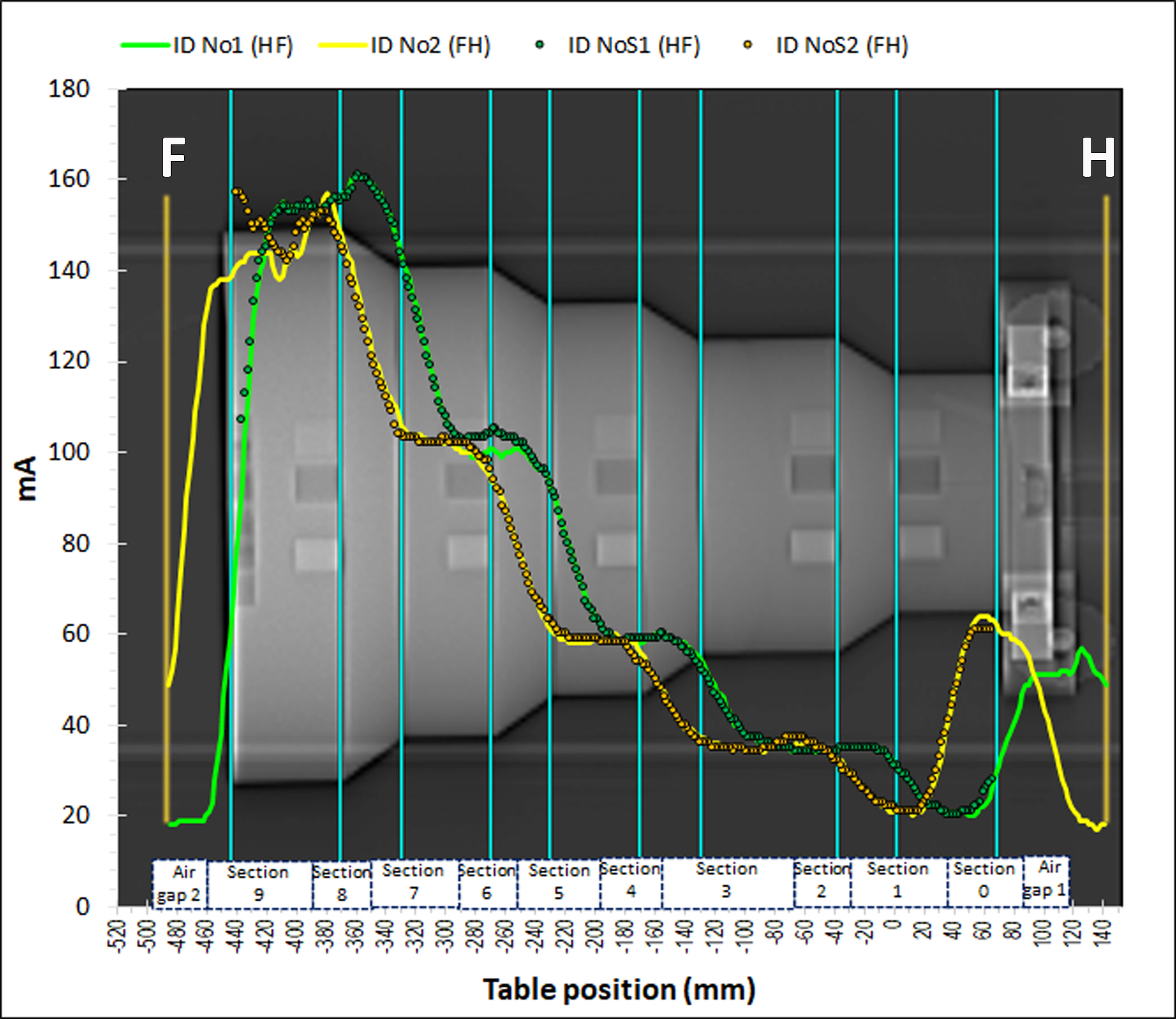

Supplement: Supplementary file 2 — Figure A1 [file ACM2-23-e13620-s005.jpg]

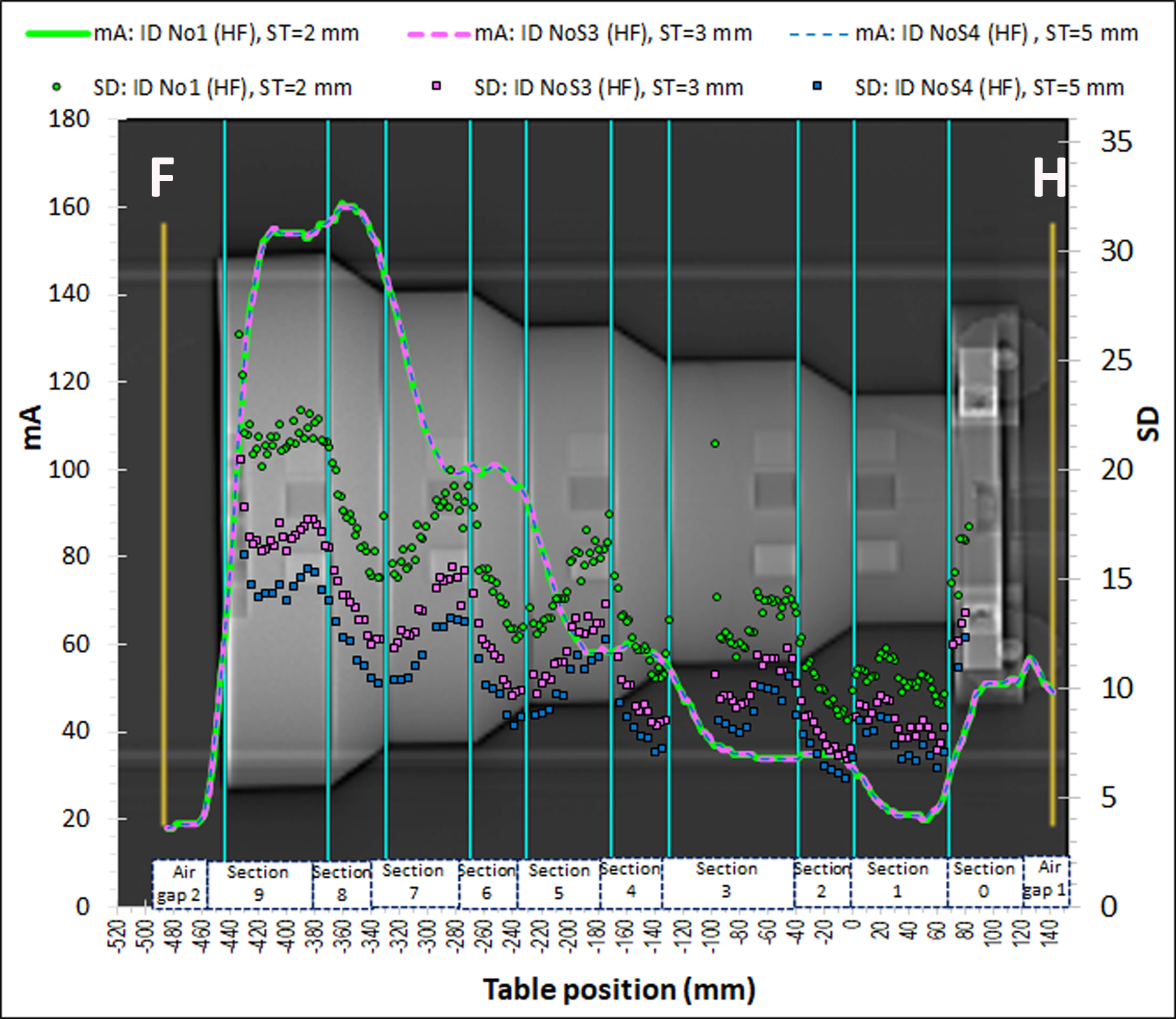

Supplement: Supplementary file 3 — Figure A2 [file ACM2-23-e13620-s008.jpg]

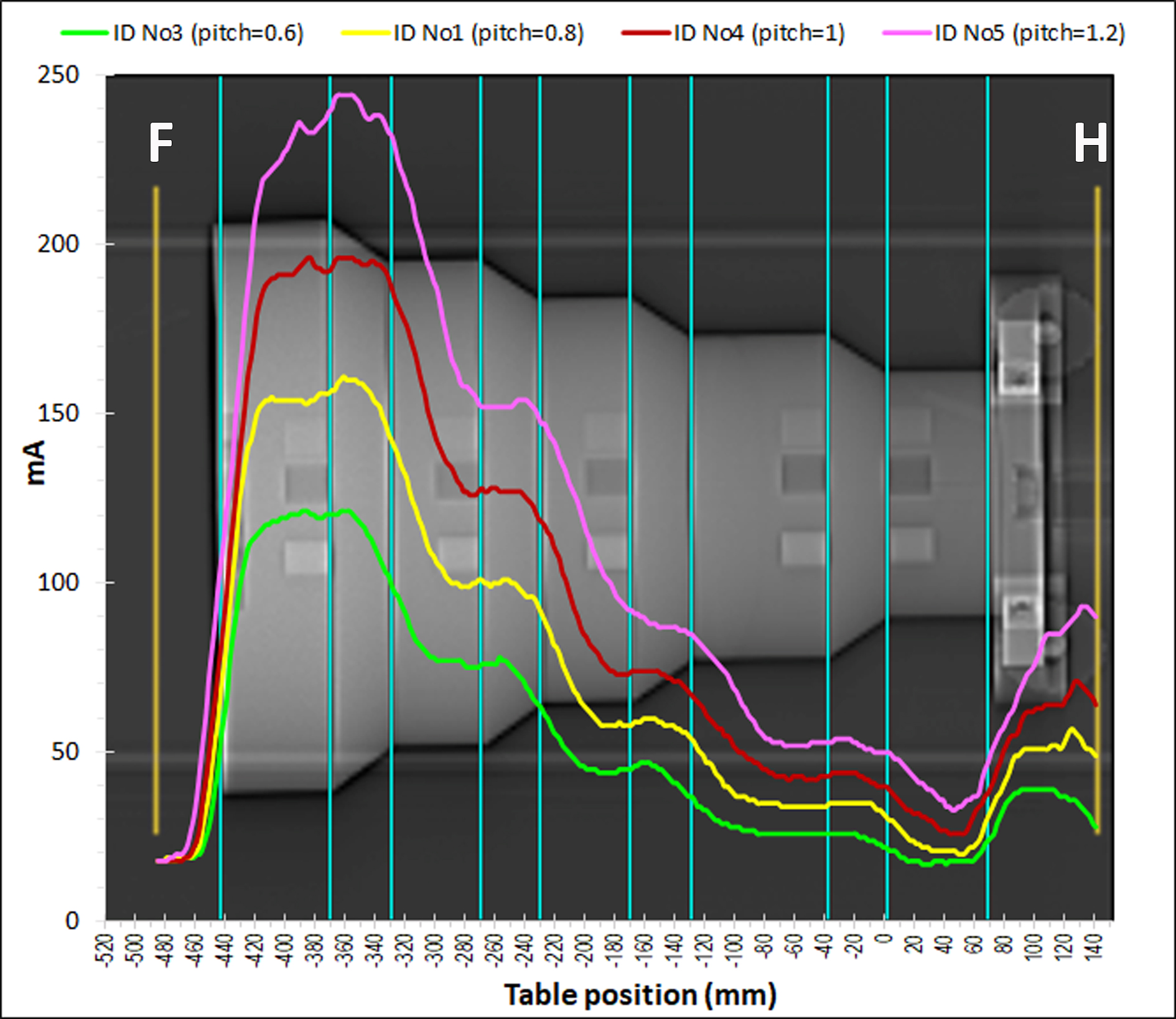

Supplement: Supplementary file 4 — Figure A3 [file ACM2-23-e13620-s006.jpg]

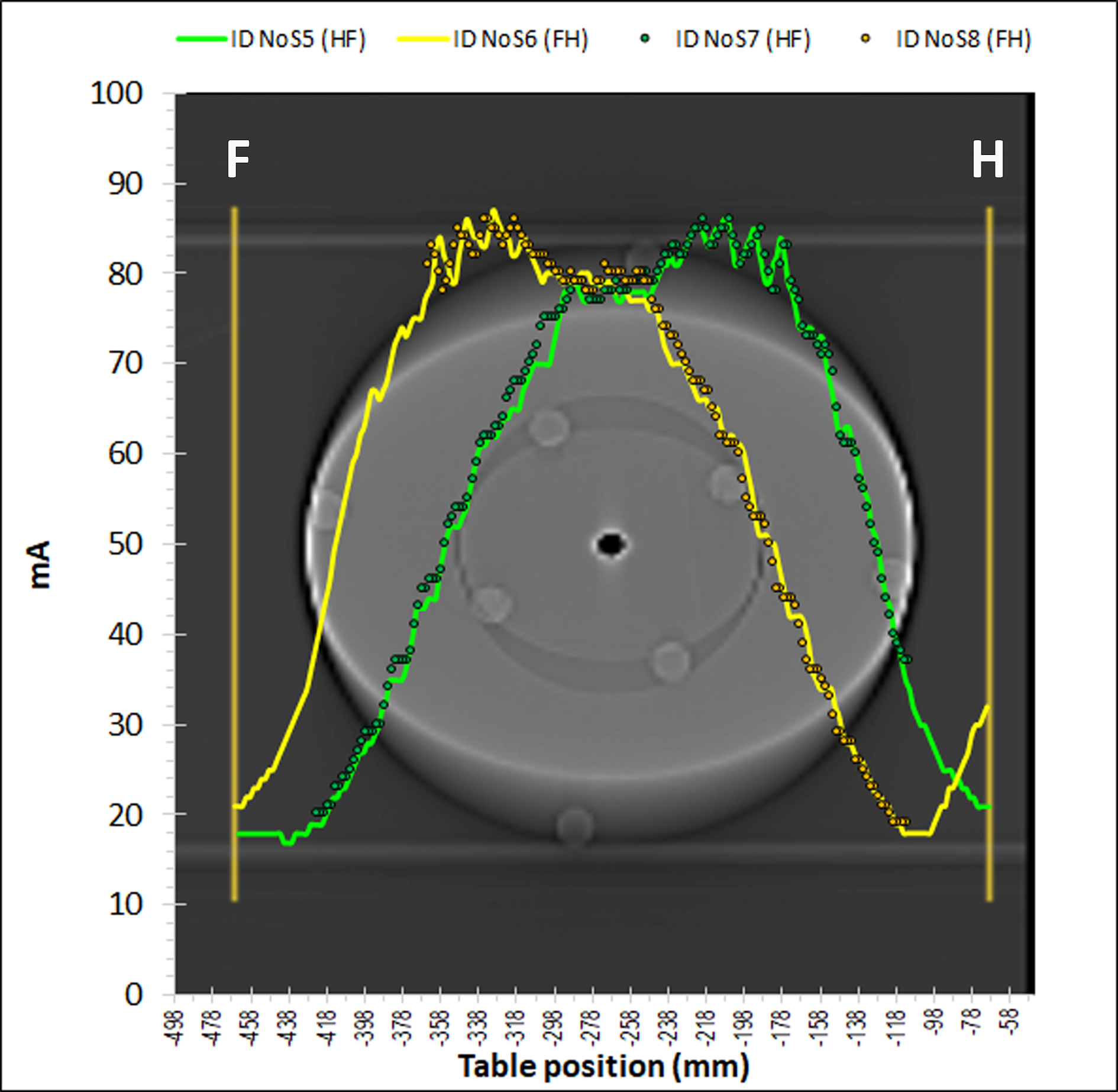

Supplement: Supplementary file 5 — Figure A4 [file ACM2-23-e13620-s001.jpg]

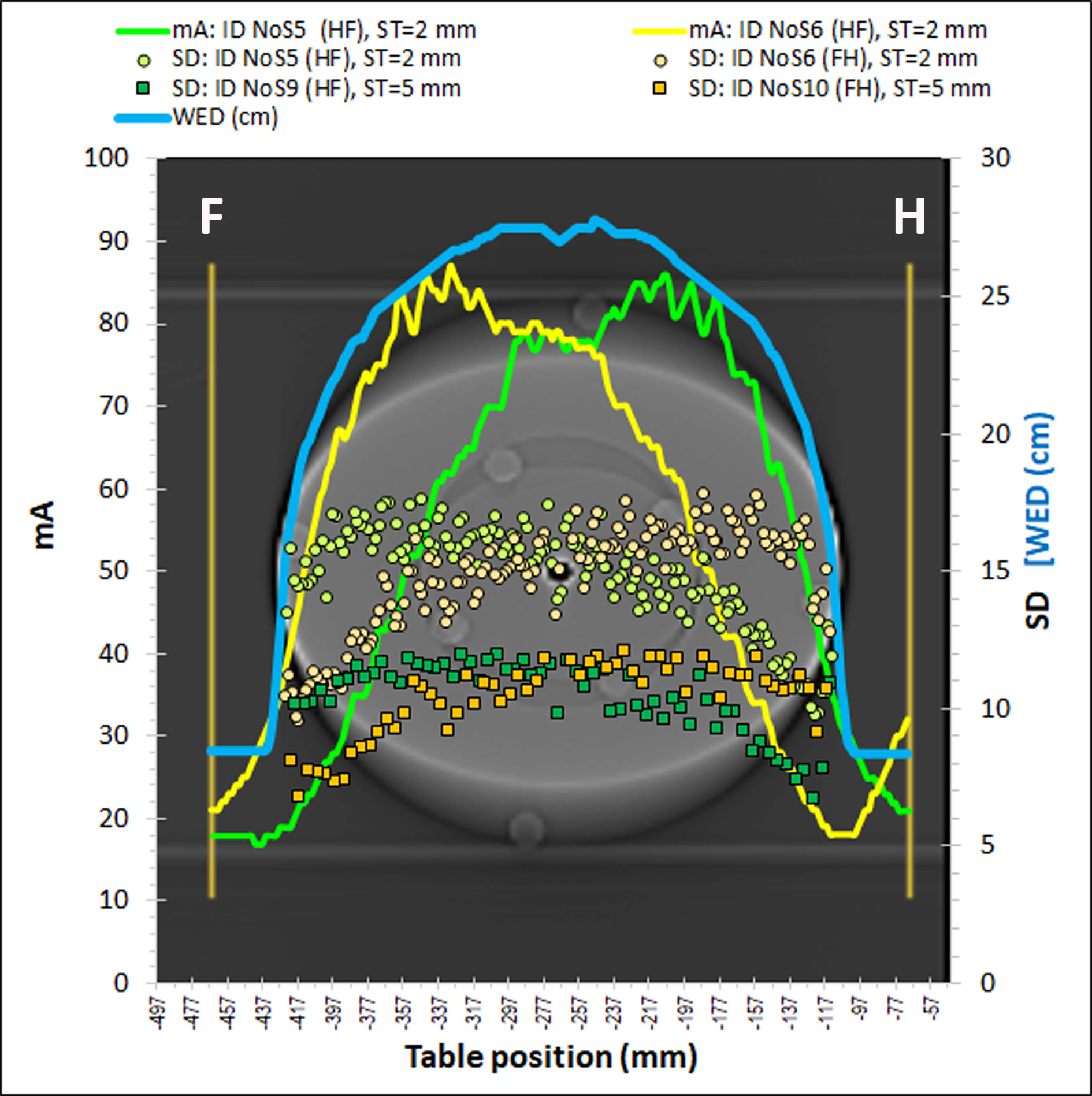

Supplement: Supplementary file 6 — Figure A5 [file ACM2-23-e13620-s004.jpg]

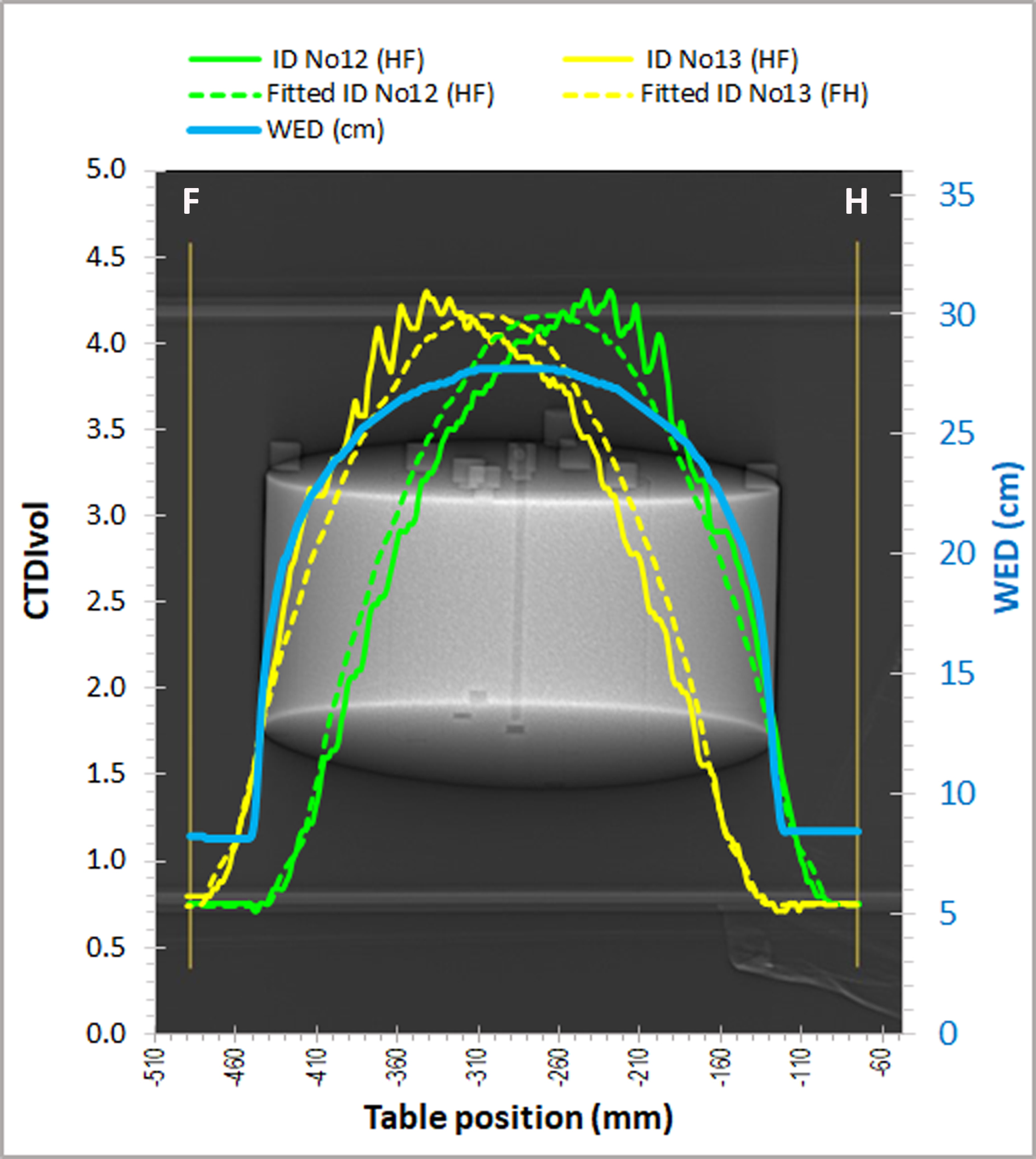

Supplement: Supplementary file 7 — Figure A6 [file ACM2-23-e13620-s007.jpg]

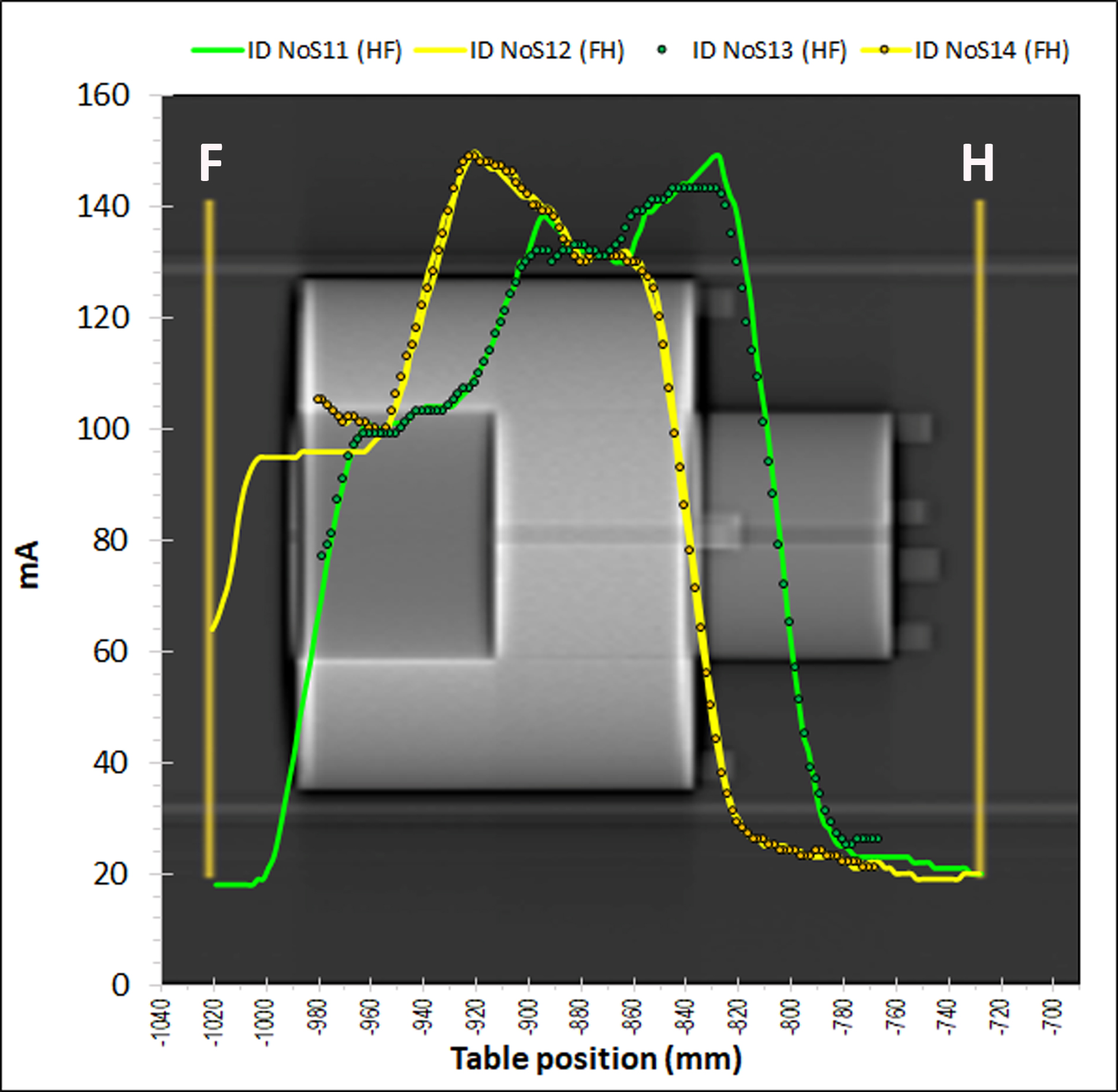

Supplement: Supplementary file 8 — Figure A7 [file ACM2-23-e13620-s002.jpg]
